# Co-Expression Network Models Suggest that Stress Increases Tolerance to Mutations

**DOI:** 10.1038/srep16726

**Published:** 2015-11-16

**Authors:** Sonja Lehtinen, Jürg Bähler, Christine Orengo

**Affiliations:** 1 Department of Infectious Disease Epidemiology, Imperial College, St Mary’s Campus, Norfolk Place, London W2 1PG, UK; 2 University College London, ISMB, London, WC1E 6BT, UK; 3 University College London, GEE, London, WC1E 6BT, UK

## Abstract

Network models are a well established tool for studying the robustness of complex systems, including modelling the effect of loss of function mutations in protein interaction networks. Past work has concentrated on average damage caused by random node removal, with little attention to the shape of the damage distribution. In this work, we use fission yeast co-expression networks before and after exposure to stress to model the effect of stress on mutational robustness. We find that exposure to stress decreases the average damage from node removal, suggesting stress induces greater tolerance to loss of function mutations. The shape of the damage distribution is also changed upon stress, with a greater incidence of extreme damage after exposure to stress. We demonstrate that the change in shape of the damage distribution can have considerable functional consequences, highlighting the need to consider the damage distribution in addition to average behaviour.

Robustness, the ability to maintain biological function in the face of perturbation, is considered a fundamental property of evolvable complex systems^1^. Micro-organisms achieve robustness to environmental perturbations (for example changes in nutrient levels, pH or temperature) through a large-scale re-arrangement of gene regulation known as the *stress response*. In broad terms, this response consists of shifting resources away from growth and proliferation and towards protective mechanisms^2^,^3^. These transient changes are accompanied by longer term effects: exposure to stress results in higher tolerance against future perturbation for both the same and different types of stress^4^. While this cross-protection effect between *environmental* stresses is well documented, less is known about the relationship between the stress response and robustness to *genetic* perturbations (‘mutational robustness’).

There is some evidence to suggest environmental and mutational robustness are mediated via similar mechanisms: a gene’s contribution to mutational robustness correlates with its effect on environmental robustness^5^. Cross-protection could thus extend from environmental perturbations to genetic perturbations. Indeed, exposure to stress has been shown to decrease the phenotypic effects of some mutations in *Caenorhabditis elegans*^6^. In *Saccharomyces cerevisiae*, a systematic study of single-gene deletions found that the average effect of gene deletion on growth rate was smaller in a number of stress-inducing environments than under standard conditions, suggesting that the stress response may indeed protect against the effects of mutation^7^. Similar results have also been reported in *Escherichia coli*^8^. Furthermore, as exposure to stress is associated with an increased rate of mutation^9^, an increase in mutational robustness could be beneficial to stressed cells. Additionally, there is evidence to suggest that stress acts as a driver of evolutionary innovation^2^. Although it seems somewhat counter intuitive, increased mutational robustness can, under some circumstances, promote greater evolvability^10^. It is therefore interesting to ask whether exposure to stress also increases mutational robustness.

Network models are well established tools for studying robustness in complex systems^11^. A network is a representation of some entities of interest (*nodes*) and the relationships between them (*edges*). Network robustness is typically studied by removing a proportion of a network’s nodes (or edges) and measuring the change in some network property or properties relating to overall network connectivity. The tacit assumption is that these properties capture how well the network is able to perform its function.

In physical protein interaction networks (where edges indicate protein binding), there is evidence to suggest that node removal captures the consequences of loss of function mutations: high degree nodes have been shown to (a) considerably disrupt measures of network connectivity when removed from the network, and (b) correspond to genes likely to be essential^12^. To some extent, this centrality-lethality relationship may be accentuated by biases in interactome mapping: high-throughput protein interaction detection techniques have been found to favour highly expressed and highly conserved proteins^13^, both of which are also likely features of essential proteins. Indeed, the centrality-lethality correlation has been found to be missing in high quality physical interaction networks mapped using techniques less likely to exhibit these types of biases^14^. Nevertheless, the effect is unlikely to be entirely caused by bias because it has been documented in a wide range of networks^15^, including co-expression networks (see below)^16^, which are not susceptible to the same mapping biases as physical interaction networks.

Much of the emphasis in the literature, particularly for work on real-world networks, has been on comparing random node failure to targeted removal of central nodes. In general terms, complex real-world networks have been found to be resilient against random failure, but vulnerable in the face of targeted attack^17^,^18^,^19^. Work on random node failure has focused almost entirely on *average* behaviour: to our knowledge, only a single study has discussed the *range* of the damage level that can be caused by random node removal. Trajanovski *et al.* used repeated realisations of random node removal to generate a probability density function for the damage in a number of theoretical network models and real-world networks^20^. The authors reported that two networks with a similar average robustness may differ considerably in the range of the expected network damage. Trajanovski *et al.* did not focus in detail on the shape of the probability density function. Given the stochasticity of biological processes, we speculated that the shape of this distribution may have substantial consequences in terms of the cell’s ability to function and survive.

In this work, we use fission yeast (*Schizosaccharomyces pombe*) co-expression networks previously derived from expression data from different yeast strains before and after exposure to oxidative stress^21^, to study the effect of stress on mutational robustness. A co-expression network represents similarities in genes’ patterns of expression. As genes involved in the same function are likely to be co-regulated and therefore co-expressed, these networks capture functional associations between proteins^22^. Because they are not affected by the same biases in interactome mapping as physical interaction networks, co-expression networks provide a valuable complementary approach. This is particularly pertinent when studying the effects of different cellular environments or conditions: there is little data mapping physical interactions under different conditions whereas condition-specific co-expression data is available. Co-expression networks therefore provide a unique opportunity to study condition dependent changes in protein network topology.

## Results

### Damage distribution shape is different in SF and ER networks

**Figure 1:**
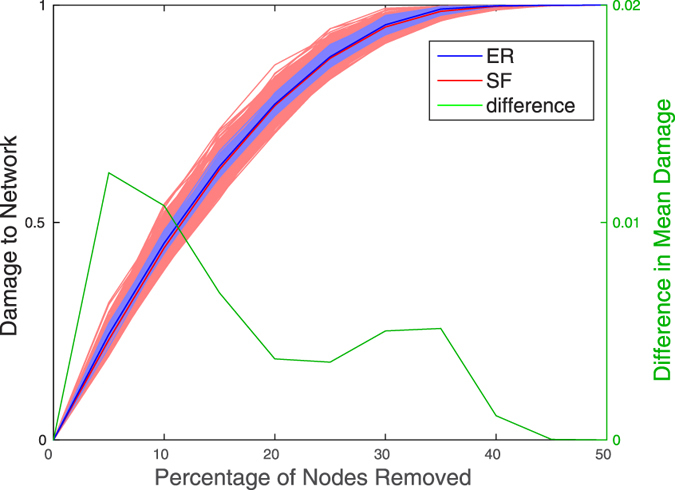
Damage to the network (measured as decrease in efficiency) in response to removal of an increasing proportion of the nodes in a SF and ER network. Each dotted line represents one realisation of random node removal, with a total of 500 realisation for each network. The solid lines represent average behaviour across realisations. The difference in average damage (ER minus SF) is shown in green, corresponding to the y-axis on the right.

First we compared the damage to scale-free (SF) Barabasi-Albert graphs^23^ and Erdős-Rényi (ER) random graphs under random node removal. We examined damage to the network, measured in terms of decrease in efficiency (a network property capturing the global connectivity of the network - see Methods), after removal of an increasing proportion of the nodes in random order. To produce an estimate of the damage probability density function (or ‘damage distribution’), we repeated this procedure 500 times (500 ‘realisations’) (Fig. 1). As reported previously, SF networks are, on average, less damaged by node removal. Importantly, however, the shape of the damage distribution is different, with greater variance and skewness in the SF network. (Skewness is a measure of the asymmetry of a distribution where a positive value indicates the data is spread to the left of the mean, and is given by *s* = *E*(*x* − *μ*)^3^/*σ*^3^ where *μ* and *σ* are the mean and standard deviation of *x* and *E*(*t*) represents the expected value of *t*).

**Figure 2:**
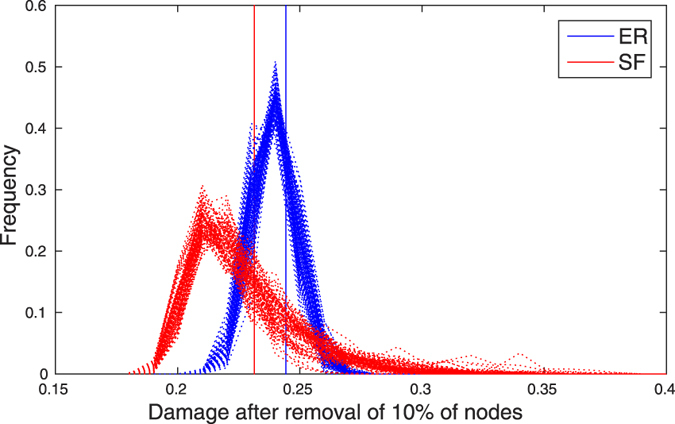
Distribution of the damage to the network (measured in terms of decrease in efficiency) after removal of 10% of the nodes for 500 realisations of random node removal for SF and ER networks. Each dotted line corresponds to the distribution of damage for a single network. The vertical lines indicate average damage across the 100 networks and 500 realizations.

For example, at 10% node removal, the mean damage is 0.23 vs 0.24, the variance is 4.3 × 10^−4^ vs 6.5 × 10^−5^ and the skewness is 0.99 vs 0.10 (SF and ER, respectively). This pattern is preserved up to almost complete breakdown of the network (see Supplementary Table 1 for details). In order to verify that this behaviour is not a property of these particular network realisations, we repeated the 500 removal realisations in 100 networks of each type (Fig. 2).

### Removal of essential genes causes greater loss of efficiency than removal of non-essential genes

Using node removal to model loss of function mutation assumes that the change in network properties (in our case network efficiency) reflects the phenotypic consequences of the mutation. To verify this assumption for the co-expression networks used in this study, we compared the loss of efficiency caused by removal of essential and non-essential genes from the pre-stress network, using a set of essential (n = 1092) and non-essential (n = 3428) genes downloaded from PomBase^24^. Removal of essential genes did indeed cause greater average damage than removal of non-essential genes (4.89 × 10^−4^ vs 4.31 × 10^−4^, p = 0.0047, Wilcoxon rank-sum), confirming node removal can indeed be used as a model for loss of function mutation.

### Damage distribution shape is different in yeast co-expression networks after exposure to stress

**Figure 3:**
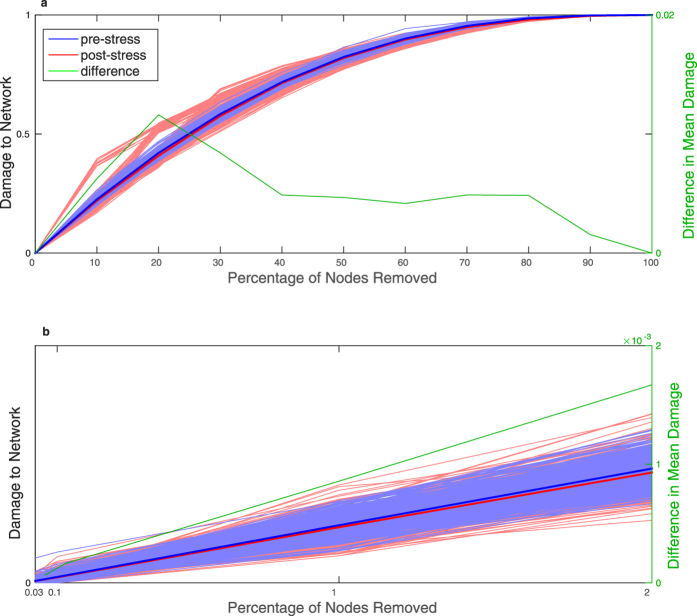
Damage in response to removal of an increasing proportion of the nodes in fission yeast co-expression networks before and after exposure to stress. The top panel (**a**) shows results for the full simulation (up to removal of all nodes), while the bottom panel (**b**) focuses on the removal of a smaller number of nodes (up to 2%). Damage is measured as the decrease in network efficiency. Each dotted line represents one realisation of random node removal, with a total of 500 realisation for each network. The solid lines represent average behaviour across realisations. The difference in average damage (pre-stress minus post-stress) is shown in green, corresponding to the y-axis on the right. The co-expression networks were derived from expression data from different yeast strains before and after exposure to oxidative stress^21^.

To study the effect of stress on the mutational robustness of fission yeast, we examined the damage caused by random node removal in the co-expression networks before and after exposure to oxidative stress (Fig. 3). Across 500 realisations, the average damage was smaller after exposure to stress (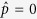 as estimated by bootstrapping; see Methods; 95% CI [0, 3.7 × 10^−5^]), suggesting stress increases mutational robustness.

**Figure 4:**
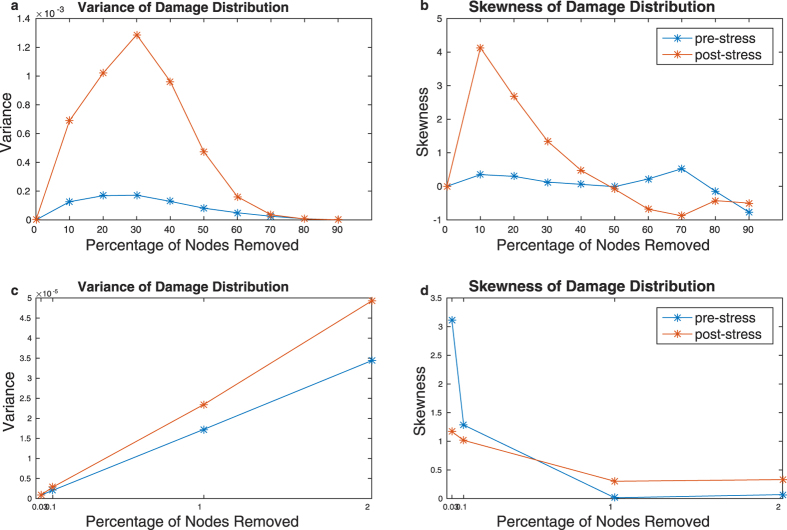
Variance (left) and skewness (right) of the distribution of damage to the network (i.e. decrease in efficiency) in response to random node removal. This distribution arises from 500 realizations depicted in Fig. 3. The top panels (**a**,**b**) show results for the full simulation (up to removal of all nodes), while the bottom panels (**c**,**d**) focus on the removal of a smaller number of nodes (up to 2%). These changes in the damage distribution indicate that while on average, loss of function mutations have a smaller effect after exposure to stress, they will occasionally lead to catastrophic failure in the post-stress network.

The effect of stress on the mean damage, although significant, is small in numerical terms (see Supplementary Tables 2 and 3). However, as with the ER and SF network models, there is a clear difference in the shape of the damage distribution: as illustrated in Fig. 4, both the variance and skewness (from removal of 1% of the nodes to removal of 50% of the nodes) of the distribution are greater after exposure to stress. Thus, after exposure to stress, while most of the time random loss of function mutations have a smaller effect on the network than prior to the stress, they occasionally lead to catastrophic damage.

For the removal of a small number of nodes (2 and 6, corresponding to 0.03% and 0.1% of the nodes in the network), the skewness of the distribution was greater *prior* to the stress. While this could reflect a genuine difference in the shape of the damage distribution when removing a smaller number of nodes, this behaviour could also result from uncertainty related to the relatively small number of realisations (n = 500): if nodes associated with catastrophic damage when removed are relatively rare, 500 realisations may not be enough to fully explore the damage distribution for removal of a small number of nodes. In order to address this, we examined the effect of removing a single node, for all nodes in the network. This replicated our earlier results: the average damage is smaller after exposure to stress (4.1 × 10^−4^ vs 3.9 × 10^−4^ before and after stress, respectively), while the variance and skewness both increase (3.1 × 10^−7^ vs 4.8 × 10^−7^ for variance and 2.0 vs 3.5 for skewness).

### Stress-induced changes are not explained by a change in degree distribution

The different robustness of the ER and SF networks are often explained in terms of their degree distributions^17^. However, the pre- and post-stress networks’ degree distributions appear quite similar (see Supplementary Figure 1). We therefore sought to establish whether the changes in the degree distribution were enough to explain the change to the damage distribution.

We generated control networks with identical expected degree distributions to the co-expression networks (see Methods), but otherwise differing network structure, and examined the shape of the damage distribution for 500 realisations of removing 10% of the nodes in these control networks (see Supplementary Figure 2). Averaging across 20 control networks, the mean damage was 0.215 and 0.211 (pre- and post-stress, respectively), the variance 1.17 × 10^−4^ and 1.68 × 10^−4^ and skewness 0.03 and −0.01. Thus, based on the change in degree distribution alone, we would expect a smaller increase in variance and a *decrease* in skewness. We conclude that the changes observed in the shape of the co-expression network damage distribution are not explained by the change in degree distribution.

### Damage distribution shape has functional consequences

**Figure 5:**
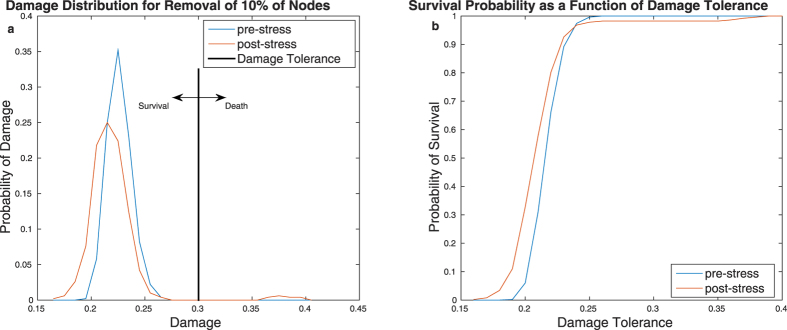
Illustration of how different network structures may be beneficial in different environments. The left hand panel (**a**) shows the damage distribution *p*(*d*) for 10% node removal for the pre- and post-stress networks. The damage tolerance *t*, capturing the level of network damage the cell can sustain before dying is also illustrated. The right hand panel (**b**) shows the relationship between overall survival probability 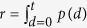 and the damage tolerance for both networks. For high damage tolerance (i.e. unchallenging environments where the cell is able to survive despite a high level of network damage), the pre-stress network has a higher overall survival probability than the post-stress network because of the occasional catastrophic failures of the post-stress network. However, if the damage tolerance is low, the pre-stress network performs better than the post-stress network. Thus, the shape of the damage distribution can have important consequences for the cell.

To illustrate the potential consequences of the differences in the shape of the damage distribution for the cell, we assume a cell’s survival probability depends on the permissiveness of its environment. In unchallenging environments, the cell has a high damage tolerance: the network can sustain a large amount of damage before the survival of the cell is impaired. In difficult environments, on the other hand, damage tolerance is lower: a smaller amount of network damage will impair the cell’s survival. As proof of concept, we created a simple model in which the cell only survives if the network damage is below threshold *t* (Fig. 5). The overall probability of survival *r* thus depends on the damage distribution *p*(*d*):


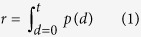


As shown in Fig. 5, the optimal damage distribution depends on the damage tolerance: at high damage tolerance (unchallenging environments), the pre-stress network has a greater overall probability of survival because in the post-stress cells, the occasional catastrophic failures fall beyond the tolerated damage. At low damage tolerance (difficult environments) however, the post-stress network performs better than the pre-stress network because very low damage is more common in the post-stress network. This illustrates how the shape of the damage distribution can have meaningful consequences for the cell’s ability to function and survive.

## Discussion

Our network model of mutational robustness suggests that exposure to stress makes cells more resistant to loss of function mutations. Indeed, it is known that various forms of stress increase resistance to U.V. radiation and other DNA-damaging agents in both prokaryotic and eukaryotic single-celled organisms^25^. This effect is generally attributed to changes in the cell’s DNA repair mechanism. Our results, however, raise the possibility that stress-induced protection from DNA-damaging agents could instead arise from increased tolerance to DNA damage conferred by wider changes to the cell’s functional organisation. This idea is supported by the observation that in budding yeast (*Saccharomyces cerevisiae*), few, if any, of the genes necessary for DNA repair are induced by exposure to DNA-damaging agents (including oxidative stress)^26^. This suggests the increased resistance to further insults produced by exposure to these stressors is mediated by mechanisms other than improved DNA repair.

The difference in average damage from node removal, though statistically significant, is relatively small. There is, however, a clear change in the shape of the damage distribution: the post-stress distribution is skewed towards low damage and has a longer tail at higher damage values. In other words, most of the time the post-stress network experiences a relatively low level of damage while occasionally succumbing to very high damage levels.

We find that changes in the degree distribution of the co-expression networks after exposure to stress do not fully explain the change in damage distributions. A significant body of previous work suggests that other properties, such as clustering coefficient, degree-degree correlation and local structure (short loops and graphlets), provide further insight into the biological function of the network. For example, degree-degree correlation has been shown to play a role in network tolerance to node removal in both network models^27^ and protein networks^28^,^29^. It is therefore possible that stress-induced changes in these other properties could predict the change in damage distribution.

We have previously documented changes in the modular structure of these co-expression networks in response to stress: sets of genes become more tightly co-regulated but co-regulation between these modules is decreased^21^. Whether this increase in network modularity accounts for the increase in robustness to node removal is unclear: while there is evidence pointing to a negative association between modularity and robustness in dynamic network models^30^,^31^, the relationship between modularity and static network models remains less well studied. Our previous work also suggested a stress-induced decrease in centrality of genes involved in various cellular processes such as ion transport and metabolism - the biological interpretation of the change in damage distribution upon node removal may therefore relate to changes in the importance of these cellular processes.

As discussed previously, exposure to stress is associated with a higher rate of mutation^9^. Increased mutational robustness would therefore allow cells to tolerate the higher frequency of deleterious mutations but also enable stressed cells to explore a greater range of new, potentially adaptive, phenotypes. Furthermore, exposure to stress is associated with higher levels of heterogeneity within a population of cells, making it more likely for at least part of the population to survive a change in conditions (‘bet hedging’)^2^. It is therefore possible that the increased variance of the damage distribution relates to the increase in heterogeneity under stressed conditions.

We have also demonstrated that the change in shape of the damage distribution can have functional consequences for the cell: which network has the better overall chance of survival depends on the level of network damage tolerated before the survival of the cell is impaired. This suggests another possible interpretation for the change in network behaviour: it is tempting to speculate that in stress-inducing environments survival may indeed be impaired at a lower level of network damage because of the additional burden imposed by the stress. In other words, it is plausible that cells in stressed environments have lower damage tolerance. The change in the damage distribution may therefore represent a strategy optimising overall survival probability of the cell in face of random mutations. Whether or not this interpretation is correct, we have demonstrated the importance of considering the shape of the distribution of damage caused by random node removal instead of simply focusing on worst-case or average behaviour.

## Methods

### Damage measure

Damage to the network was measured in terms of decrease in network efficiency *e*, given by:


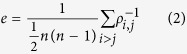


with *ρ* indicating the length of the shortest path between nodes *i* and *j*. The change in network efficiency in response to node removal was measured by the normalized change in efficiency 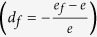, where *e*_*f*_ is the efficiency after removal of fraction *f* of the nodes. Adopting the approach taken by Trajanovski *et al.*^20^, removal of a node from the network was modelled by deletion of all the node’s edges instead of removal of the node itself. Thus, when computing *e*_*f*_, *n* remains equal to the number nodes in the original network. Using this approach, the damage measure (*d*_*f*_) is constrained between 0 (no damage) and 1 (fully disconnected network).

### Theoretical network models

Resilience to node removal was compared in scale-free (SF) Barabasi-Albert graphs^23^ and Erdős-Rényi (ER) random graphs. Network generation, node removal and path calculations were all implemented using the NetworkX package for Python.

SF networks were generated according to the preferential attachment model: the network is initialized with *m* nodes and grown one node at the time, until a network with *n* nodes is reached. Each new node attaches to *m* of the existing nodes. The probability of attaching to existing node *i* (*τ*_*i*_) is proportional to the degree of *i* (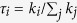, where *k*_*i*_ is the degree of *i*).

ER networks were generated by initializing a network with *n* nodes and connecting each pair of nodes with probability *p*.

In the work presented here, we used values *n* = 1000 and *m* = 2, giving a SF network with 1000 nodes and 1996 edges. This corresponds to a *p* of 0.004 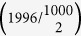 for ER network generation. Because ER network generation is a probabilistic process, there was slight variation in the number of edges in the ER network.

### Co-expression networks

Gene co-expression networks were constructed using gene expression data from different genetic strains in fission yeast, before and after exposure to oxidative stress (0.5 mM hydrogen peroxide, H_2_O_2_). Spearman correlation coefficients were computed across the genetic variants for each gene pair, before and after exposure to stress. To generate the networks, 4 × 10^4^ gene pairs with the highest significant (p < 0.05) correlation coefficients were considered connected, yielding an unweighted network. All nodes, even those with no edges were included in the network when modelling node removal. More details on network generation can be found in Lehtinen *et al.*^21^.

### Control networks

In order to study the effect of a network’s degree distribution on the shape of the damage distribution, we generated control networks with identical expected degree distribution but otherwise differing network structure. For a network with degree sequence *W* = (*w*_1_, *w*_2_, ..., *w*_*n*_), control networks were built by assigning an edge between nodes *i* and *j* with probability 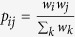 when *i* ≠ *j* and *p*_*ij*_ = 0 when *i* = *j*. The control networks were generated using the python module NetworkX.

### Statistical testing

Bootstrapping was used to estimate a p-value for the difference in damage to the two networks. Bootstrapping was chosen because the non-normality and difference in shape of the damage distributions violate the assumptions of tests such as the t-test or the Wilcoxon rank-sum test.

As we were interested in the damage for all fractions of nodes removed, we used the difference in total damage across the sampled percentages as the measure of interest. The pre-stress and post-stress realisations were randomly re-shuffled 10^5^ times and the measure of interest computed for each permutation, giving a null distribution from which the p-value was estimated. The confidence interval for the p-value was calculated using the binomial distribution. For comparison, p-values from the Wilcoxon rank-sum test are included in Supplementary Table 2 and 3.

### Modelling the effects of the damage distribution

The overall probability of survival *r* was modelled as a function of the damage distribution *p*(*d*) (as estimated from the 500 node removal realisations, for 10% node removal) and a damage tolerance threshold *t*:


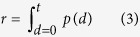


## Additional Information

**How to cite this article**: Lehtinen, S. *et al.* Co-Expression Network Models Suggest that Stress Increases Tolerance to Mutations. *Sci. Rep.* **5**, 16726; doi: 10.1038/srep16726 (2015).

## Supplementary Material

Supplementary Information

## References

[b1] Kitano H. Biological robustness. Nat. Rev. Genet. 5, 826–837 (2004). 15520792 10.1038/nrg1471

[b2] López-Maury L., Marguerat S. & Bähler J. Tuning gene expression to changing environments: from rapid responses to evolutionary adaptation. Nat. Rev. Genet. 9, 583–593 (2008). 18591982 10.1038/nrg2398

[b3] Saito H. & Posas F. Response to hyperosmotic stress. Genetics 192, 289–318 (2012). 23028184 10.1534/genetics.112.140863 PMC3454867

[b4] Berry D. B. & Gasch A. P. Stress-activated genomic expression changes serve a preparative role for impending stress in yeast. Mol. Biol. Cell 19, 4580–4587 (2008). 18753408 10.1091/mbc.E07-07-0680 PMC2575158

[b5] Lehner B. Genes confer similar robustness to environmental, stochastic, and genetic perturbations in yeast. PloS One 5, e9035 (2010). 20140261 10.1371/journal.pone.0009035 PMC2815791

[b6] Casanueva M. O., Burga A. & Lehner B. Fitness trade-offs and environmentally induced mutation buffering in isogenic C. elegans. Science 335, 82–85 (2012). 22174126 10.1126/science.1213491

[b7] Jasnos L. *et al.* Interactions between stressful environment and gene deletions alleviate the expected average loss of fitness in yeast. Genetics 178, 2105–2111 (2008). 18430936 10.1534/genetics.107.084533 PMC2323800

[b8] Kishony R. & Leibler S. Environmental stresses can alleviate the average deleterious effect of mutations. J. Biol. 2, 14 (2003). 12775217 10.1186/1475-4924-2-14 PMC193686

[b9] Galhardo R. S., Hastings P. J. & Rosenberg S. M. Mutation as a stress response and the regulation of evolvability. Crit. Rev. Biochem. Mol. 42, 399–435 (2007). 10.1080/10409230701648502 PMC3319127 17917874

[b10] Wagner A. The role of robustness in phenotypic adaptation and innovation. Proc. R. Soc. B 279, 1249–1258 (2012). 10.1098/rspb.2011.2293 PMC3282381 22217723

[b11] Callaway D. S., Newman M. E. J., Strogatz S. H. & Watts D. J. Network robustness and fragility: Percolation on random graphs. Phys. Rev. Lett. 85, 5468–5471 (2000). 11136023 10.1103/PhysRevLett.85.5468

[b12] Jeong H., Mason S. P., Barabasi A. L. & Oltvai Z. N. Lethality and centrality in protein networks. Nature 411, 41–42 (2001). 11333967 10.1038/35075138

[b13] Von Mering C. *et al.* Comparative assessment of large-scale data sets of protein-protein interactions. Nature 417, 399–403 (2002). 12000970 10.1038/nature750

[b14] Yu H. *et al.* High-quality binary protein interaction map of the yeast interactome network. Science 322, 104–110 (2008). 18719252 10.1126/science.1158684 PMC2746753

[b15] Raman K., Damaraju N. & Joshi G. The organisational structure of protein networks: revisiting the centrality–lethality hypothesis. Syst. Synth. Biol. 8, 73–81 (2014). 24592293 10.1007/s11693-013-9123-5 PMC3933631

[b16] Carlson M. R. J. *et al.* Gene connectivity, function, and sequence conservation: predictions from modular yeast co-expression networks. BMC Genomics 7, 40 (2006). 16515682 10.1186/1471-2164-7-40 PMC1413526

[b17] Albert R., Jeong H. & Barabasi A.-L. Error and attack tolerance of complex networks. Nature 406, 378–382 (2000). 10935628 10.1038/35019019

[b18] Holme P., Kim B. J., Yoon C. N. & Han S. K. Attack vulnerability of complex networks. Phys. Rev. E 65, 056109 (2002). 10.1103/PhysRevE.65.056109 12059649

[b19] Newman M. E. J. The structure and function of complex networks. SIAM Review 45, 167–256 (2003).

[b20] Trajanovski S., Martn-Hernández J., Winterbach W. & Van Mieghem P. Robustness envelopes of networks. J. Complex Networks 1, 44–62 (2013).

[b21] Lehtinen S. *et al.* Stress induces remodelling of yeast interaction and co-expression networks. Mol. Biosyst. 9, 1697–1707 (2013). 23471351 10.1039/c3mb25548d

[b22] Eisen M. B., Spellman P. T., Brown P. O. & Botstein D. Cluster analysis and display of genome-wide expression patterns. Proc. Natl. Acad. Sci. USA 95, 14863–14868 (1998). 9843981 10.1073/pnas.95.25.14863 PMC24541

[b23] Barabási A.-L. & Albert R. Emergence of scaling in random networks. Science 286, 509–512 (1999). 10521342 10.1126/science.286.5439.509

[b24] Wood V. *et al.* Pombase: a comprehensive online resource for fission yeast. Nucleic Acids Res. 43, D656–D661 (2011). 22039153 10.1093/nar/gkr853 PMC3245111

[b25] Rangel D. Stress induced cross-protection against environmental challenges on prokaryotic and eukaryotic microbes. World J. Microb. Biot. 27, 1281–1296 (2011). 10.1007/s11274-010-0584-3 25187127

[b26] Birrell G. W. *et al.* Transcriptional response of saccharomyces cerevisiae to DNA-damaging agents does not identify the genes that protect against these agents. Proc. Natl. Acad. Sci. USA 99, 8778–8783 (2002). 12077312 10.1073/pnas.132275199 PMC124375

[b27] Vázquez A. & Moreno Y. Resilience to damage of graphs with degree correlations. Phys. Rev. E 67, 015101 (2003). 10.1103/PhysRevE.67.015101 12636544

[b28] Friedel C. C. & Zimmer R. Influence of degree correlations on network structure and stability in protein-protein interaction networks. BMC Bioinform. 8, 297 (2007). 10.1186/1471-2105-8-297 PMC1995226 17688687

[b29] Hao D. & Li C. The dichotomy in degree correlation of biological networks. PloS One 6, e28322 (2011). 22164269 10.1371/journal.pone.0028322 PMC3229552

[b30] Holme P. Metabolic robustness and network modularity: a model study. PLoS One 6, e16605 (2011). 21311770 10.1371/journal.pone.0016605 PMC3032788

[b31] Tran T.-D. & Kwon Y.-K. The relationship between modularity and robustness in signalling networks. J. R. Soc. Interface 10, 20130771 (2013). 24047877 10.1098/rsif.2013.0771 PMC3785844

