# Supplementary material for: Co-Expression Network Models Suggest that Stress Increases Tolerance to Mutations
Source: Sci Rep. 2015 Nov 16;5:16726. doi: 10.1038/srep16726 (PMC4644955; doi:10.1038/srep16726)
Supplement: Supplementary Information [file srep16726-s1.pdf]

Supplementary Information

Manuscript title: Co-Expression Network Models Suggest that Stress Increases Tolerance to Mutations.
Authors: S. Lehtinen, J. Bähler, C. Orengo.

Nodes Removed	10%	20%	30%	40%	50%	60%	70%	80%	90%
Mean ER	0.243	0.452	0.629	0.772	0.881	0.955	0.991	0.998	1.000
Mean SF	0.230	0.442	0.622	0.769	0.878	0.950	0.986	0.997	1.000
P-value	8.98E-40	4.07E-22	2.52E-10	9.01E-05	4.02E-05	1.14E-09	6.68E-32	4.73E-19	9.00E-05
Variance ER	6.45E-05	1.00E-04	1.08E-04	1.05E-04	9.21E-05	6.14E-05	1.30E-05	2.00E-07	6.65E-09
Variance SF	4.34E-04	8.62E-04	8.95E-04	7.64E-04	4.85E-04	2.31E-04	5.31E-05	3.51E-06	4.61E-08
Skewness ER	0.10	0.20	0.13	-0.09	0.11	-0.09	-0.88	-1.54	-0.96
Skewness SF	0.99	0.77	0.50	0.29	0.37	0.02	-0.54	-1.42	-2.47

Table 1. Supplementary Table 1: Mean, variance and skewness of the distribution of damage (i.e. decrease in efficiency) to ER and SF network models in response to random node removal. The reported p-value is for the difference in the mean damage and is derived from a Wilcoxon rank-sum test.

Nodes Removed	10%	20%	30%	40%	50%	60%	70%	80%	90%
Mean Pre-Stress	0.226	0.421	0.584	0.718	0.824	0.901	0.954	0.986	0.998
Mean Post-Stress	0.220	0.409	0.576	0.714	0.819	0.897	0.949	0.981	0.997
P-value	2.10E-22	1.46E-50	1.64E-36	5.34E-11	1.71E-02	3.76E-04	4.75E-36	1.23E-106	4.49E-144
Variance Pre-Stress	1.26E-04	1.68E-04	1.72E-04	1.29E-04	8.15E-05	4.96E-05	2.54E-05	7.73E-06	2.10E-07
Variance Post-Stress	6.90E-04	1.02E-03	1.29E-03	9.63E-04	4.74E-04	1.61E-04	3.58E-05	6.42E-06	5.55E-07
Skewness Pre-Stress	0.35	0.30	0.13	0.06	-0.01	0.22	0.52	-0.15	-0.77
Skewness Post-Stress	4.13	2.67	1.35	0.48	-0.08	-0.68	-0.88	-0.42	-0.50

Table 2. Supplementary Table 2: Mean, variance and skewness of the distribution of damage (i.e. decrease in efficiency) to the co-expression networks in response to random node removal. The reported p-value is for the difference in the mean damage and is derived from a Wilcoxon rank-sum test.

Nodes Removed	0.03%	0.1%	1%	2%
Mean Pre-Stress	0.0009	0.0025	0.0243	0.0482
Mean Post-Stress	0.0008	0.0023	0.0234	0.0465
P-value	0.111	0.0150	0.0010	1.09E-5
Variance Pre-Stress	8.57E-07	2.05E-06	1.73E-05	3.44E-05
Variance Post-Stress	9.16E-07	2.85E-06	2.35E-5	4.93E-05
Skewness Pre-Stress	3.1126	1.2847	0.0143	0.0691
Skewness Post-Stress	1.1673	1.0165	0.3023	0.3308

Table 3. Supplementary Table 3: Mean, variance and skewness of the distribution of damage (i.e. decrease in efficiency) to the co-expression networks in response to random node removal, for a smaller number of nodes. The reported p-value is for the difference in the mean damage and is derived from a Wilcoxon rank-sum test.

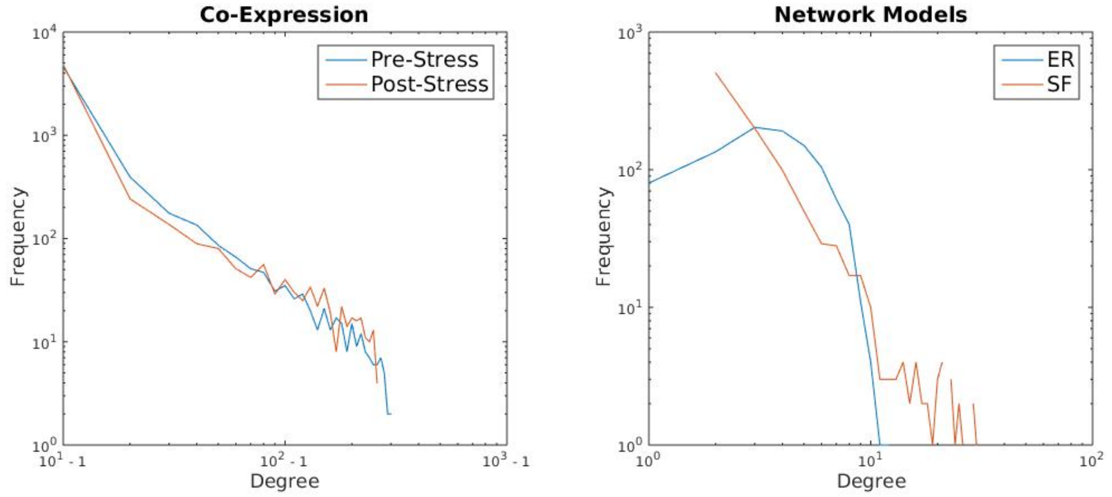

Figure 1. Supplementary Figure 1. Degree distributions in the co-expression networks (left) and the network models (right). Note the shift in the scale of the x-axis on the co-expression network plot to allow for nodes of degree zero. The y-axes of the two graphs have a different scale because the networks have a different number of nodes (5883 for the co-expression networks and 1000 for the SF and ER network models).

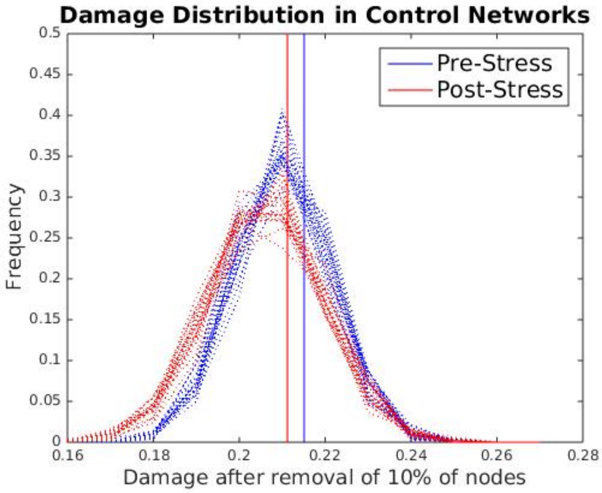

Figure 2. Distribution of network damage (i.e. decrease in efficiency) after removal of 10% of nodes for 500 realisations in 20 control networks with the same degree distribution as the pre- or post-stress co-expression network. The dotted lines represent the distribution for a single control network. The vertical lines indicate the average damage across the 20 networks and 500 realisations.
